# Hierarchical Generalized Linear Models for Multiple Groups of Rare and Common Variants: Jointly Estimating Group and Individual-Variant Effects

**DOI:** 10.1371/journal.pgen.1002382

**Published:** 2011-12-01

**Authors:** Nengjun Yi, Nianjun Liu, Degui Zhi, Jun Li

**Affiliations:** Department of Biostatistics, Section on Statistical Genetics, University of Alabama at Birmingham, Birmingham, Alabama, United States of America; University of California San Diego and The Scripps Research Institute, United States of America

## Abstract

Complex diseases and traits are likely influenced by many common and rare genetic variants and environmental factors. Detecting disease susceptibility variants is a challenging task, especially when their frequencies are low and/or their effects are small or moderate. We propose here a comprehensive hierarchical generalized linear model framework for simultaneously analyzing multiple groups of rare and common variants and relevant covariates. The proposed hierarchical generalized linear models introduce a group effect and a genetic score (i.e., a linear combination of main-effect predictors for genetic variants) for each group of variants, and jointly they estimate the group effects and the weights of the genetic scores. This framework includes various previous methods as special cases, and it can effectively deal with both risk and protective variants in a group and can simultaneously estimate the cumulative contribution of multiple variants and their relative importance. Our computational strategy is based on extending the standard procedure for fitting generalized linear models in the statistical software R to the proposed hierarchical models, leading to the development of stable and flexible tools. The methods are illustrated with sequence data in gene *ANGPTL4* from the Dallas Heart Study. The performance of the proposed procedures is further assessed via simulation studies. The methods are implemented in a freely available R package BhGLM (http://www.ssg.uab.edu/bhglm/).

## Author Summary

Complex diseases and traits are likely influenced by many common and rare genetic variants and environmental factors. Next-generation sequencing technologies have provided unparalleled tools to sequence a large number of individuals, allowing for comprehensive studies of both common and rare variants. However, detecting disease-associated rare variants and common variants of small effects poses unique statistical challenges. We propose here a comprehensive hierarchical generalized linear model framework for simultaneously analyzing multiple groups of rare and common variants and relevant covariates. The proposed hierarchical generalized linear models introduce a group effect and a genetic score for each group of variants, and jointly they estimate the group effects and the weights of the genetic scores. This framework includes various previous methods as special cases, and it can effectively deal with both risk and protective variants in a group and can simultaneously estimate the cumulative contribution of multiple variants and their relative importance. The methods are illustrated with sequence data in gene *ANGPTL4* from the Dallas Heart Study and are further assessed via simulation studies. The methods have been implemented in a freely available R package BhGLM (http://www.ssg.uab.edu/bhglm/).

## Introduction

Many common human diseases and complex traits are highly heritable and are believed to be influenced by multiple genetic and environmental factors. Genome-wide association studies (GWAS) represent a powerful way for discovering disease-associated factors and investigating the genetic architecture of complex diseases [1]. In the past few years, these studies have identified hundreds of common variants (i.e., genetic variants with minor allele frequency (MAF) >∼5%) associated with complex diseases [2]. However, the estimated effect sizes for the identified variants are small (most odds ratios are below 1.5) and explain only a small proportion of the heritability of complex diseases [2], [3], motivating research interest in finding ‘missing’ genetic factors that contribute to the remaining heritability [4], [5]. Many explanations for the missing heritability have been suggested [4], [5]; one is that many common variants with much smaller effects are yet to be detected, and another is the possible contribution of rare variants (MAF <0.5% or 1%) that are poorly captured by previous GWA genotyping arrays. Empirical studies and population genetics theory support the potentially important role of both rare variants and common variants of very small effects [6]–[10]. Several current studies have implicated association of rare variants with complex diseases and traits [11]–[18].

Next-generation sequencing technologies have provided unparalleled tools to sequence a large number of individuals in candidate genes, exomes, or even the entire genome, allowing for comprehensive studies of both common and rare variants. In addition to the common problems of handling large numbers of variants, however, detecting disease-associated rare variants and common variants of small effects poses unique statistical challenges [19], [20]. As such variants individually contain little variation, statistical methods that detect association between a single variant and disease phenotype provide low power with realistic sample sizes. Therefore, it is necessary to develop sophisticated methods that can effectively combine information across variants and assess the collective effect of multiple variants [4].

Several approaches along this line have been proposed [19], [20]. The basic procedure of these methods is to construct a linear combination of multiple variants with fixed weights to summarize the information across the variants and then estimate its association with the phenotype [21]–[25]. Different weights yield different summaries of the variants and implicate different assumptions about the relative importance of individual variants [24], [26]. Further, they implicitly assume that all variants affect phenotype in the same direction. However, there are many examples in which numerous rare variants detected in a gene or region may have disparate or even opposite effects on phenotype [4], [11]. Thus, these methods can be suboptimal if the data do not follow the underlying assumptions. Recently, several methods have been proposed to deal with variants with opposite effects [26]–[32], and to summarize the information across variants using non-linear functions [33], [34].

All the existing methods have been developed to assess only one group of variants at a time. Since common diseases are likely caused by a complex interplay among many genes and environmental factors, however, it is more appropriate to simultaneously model multiple groups of variants and covariates [19]. The joint analyses would improve the power of detecting causal effects and hence lead to increased understanding about the genetic architecture of diseases. Such methods are also advantageous for studies involving only one candidate gene, because numerous variants detected within a gene can be divided into multiple groups based on their allelic frequencies (common or rare) and functional annotations of the genomic regions they reside in (for example, non-synonymous or synonymous). It has been found in GWAS that the vast majority (80%) of associated variants fall outside coding regions, emphasizing the importance of including both coding and non-coding regions in the search for disease-associated variants [2].

We propose here a comprehensive hierarchical generalized linear model (GLM) framework for simultaneously analyzing multiple groups of rare and common variants and relevant covariates. The proposed hierarchical GLMs introduce a group effect and a genetic score (i.e., a linear combination of main-effect predictors for genetic variants) for each group of variants, and jointly estimate the group effects and the weights of the genetic scores. This framework includes various previous methods as special cases, and can effectively deal with both risk and protective variants in a group and can simultaneously estimate the cumulative contribution of multiple variants and their relative importance. The methods are illustrated with sequence data in gene *ANGPTL4* from the Dallas Heart Study, and are further assessed via simulation studies. Finally, we conclude this article by highlighting some areas of future research.

## Methods

### Hierarchical GLMs for Multiple Groups of Rare and Common Variants

Suppose that a population-based association study consists of *n* unrelated individuals, phenotyped for a continuous or discrete disease trait and genotyped for a number of rare and/or common genetic variants in one or multiple candidate genes or genomic regions. The observed values of the response variable are denoted by *y* = (*y*_1_, ···, *y_n_*). We assume that the genetic variants can be divided into *K* groups, *G_k_*, *k* = 1, ···, *K*, and the *k*-th group *G_k_* contains *J_k_* variants, where *K*≥1 and *J_k_*>1. The groups can be constructed based on candidate genes in which the variants are located and the types of the variants (e.g., common variants, rare non-synonymous or synonymous coding variants). We assume that some non-genetic variables (e.g., gender indicator, age, etc.) are also measured for each individual and will be included as covariates in the model to control for possible confounding effects.

We extend the hierarchical generalized linear model (GLM) of Yi and Zhi [26] to simultaneously fit covariates and multiple groups of rare and common variants. A generalized linear model consists of three components: the linear predictor *η*, the link function *h*, and the data distribution *p* [35], [36]. The linear predictor of individual *i* is expressed as the multiplicative form:
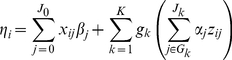
(1)where *β*_0_ is the intercept, *x_ij_* and *β_j_* represent covariate *j* and its coefficient, respectively, *z_ij_* is the main-effect predictor for individual *i* at genetic variant *j* in group *G_k_*, equaling to the number of minor alleles for an additive coding (for a rare variant, the additive coding is approximately equivalent to a dominant coding because the frequency of the minor allele is very low), the common coefficient 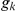 represents the *group effect* for *J_k_* variants in the *k*-th group, and the individual coefficients 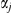 can be interpreted as the *weights* or *relative effects* of individual variants.

The common coefficient 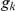 represents the association between the phenotype and the linear combination 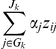 of *J_k_* individual main-effect predictors for variants in group *G_k_*. The linear combination provides a way to combine the genetic variation across the *J_k_* individual variants, referred to as *genetic score*. Therefore, the common coefficient 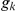 represents the cumulative importance of the *J_k_* individual variants in the *k*-th group, hence referred to as the *group effect*, and the weights 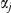, 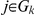, give the relative importance of the individual variants in group *G_k_*.

The mean of the response variable is related to the linear predictor via a link function *h*:
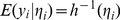
(2)The data distribution (likelihood) is expressed as
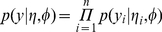
(3)where 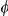 is a dispersion (or variance) parameter, and the distribution 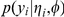 can take various forms, including Normal, Gamma, Binomial, and Poisson distributions.

Our main goal is to estimate the group effects 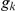 and to test the hypotheses *g_k_* = 0, *k* = 1, ···, *K*. We treat the weights 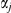
's as unknown parameters and estimate them along with the group effects and other parameters from the data. But we cannot simply use classical framework (equivalent to setting uniform distributions on the 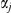
's from a Bayesian perspective), since this would result in a nonidentifiable model [37], [38]. An approach to overcoming the problem is to use an informative prior for 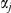. We use the following hierarchical prior distribution:
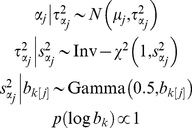
(4)where the prior means 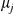 are prefixed and will be discussed in detail later, and the subscript *k*[*j*] indexes the group *k* that variant *j* belongs to.

The above hierarchical prior assumes that 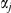 follows a scale mixture of normals with unknown variable-specific variance 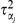. The prior distribution for 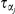 is a hierarchical formulation of the half-Cauchy distribution, which has desirable properties, such as an infinite spike at the prior mean and very heavy tails, and also facilitates efficient computation [39], [40]. An attractive feature of our hierarchical prior is that it is free of user-chosen tuning parameters and introduces group-specific parameters 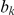 and variable-specific parameters 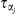 and 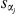. The group-specific parameters provide a way to pool the information among variables within a group and also to induce different shrinkage for different groups, while the variable-specific parameters allow different shrinkage for different variables. Yi and Zhi [26] set the scale parameters 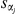 to a known value for all the weight parameters and recommended 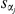 = 0.5 as default. However, it would be more reasonable to estimate the scale parameters from the data.

If the number of groups is not large, the group effects 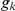 usually can be estimated classically. However, low allelic frequencies can yield very small variances for the predictors of 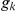, i.e., 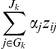, and as a result the classical procedure can result in numerically instable estimates for the group effects 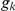. To overcome this problem, we can place a weakly informative prior on 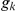 that constrains 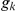 to a reasonable range [41]. We use the following hierarchical prior distribution:
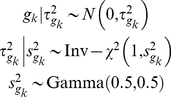
(5)This hierarchical prior distribution includes group-specific parameters 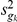, which can induce different shrinkage for different group effects 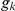. The group-specific parameters 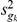 are assumed to follow a weakly informative prior Gamma(0.5, 0.5). This weakly informative prior does not strongly shrink 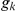 towards zero, but can constrain 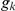 to lie in a reasonable range [41].

For the covariate effects 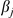, we also use the above weakly informative prior (5), i.e., 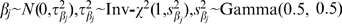. For the intercept 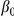 and the dispersion parameter 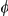, we can use any reasonable non-informative prior distributions; for example, 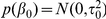 with 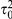 set to a large value, and 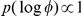.

### Model Interpretation

Our hierarchical GLMs include multiplicative parameters, a common coefficient 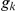 for a group of variants and a weight parameter 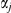 for each variant. As explained earlier, the common coefficient 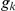 represents the overall association of the *J_k_* individual variants in group *k* with the disease. In our hierarchical model, the multiplicative term 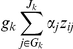 can be expressed as 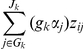, and thus the predictor *z_ij_* ultimately gets the coefficient 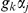, which represents the main effect of that variant. The coefficient 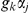 is affected by the prior mean of 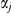. Therefore, we define the adjusted main effects as 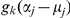, which represent the effects of individual variants.

For the multiplicative model to be useful, we need informative prior distributions on the multiplicative parameters that allow us to distinguish between the group effects and the individual weights. The prior means 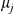 and the variances 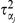 in the normal prior distributions of the weights 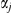 (i.e., 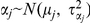) are the key components to interpret our hierarchical model. The variances 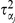 directly control the amount of shrinkage for 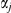. If 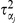 = 0, the coefficient 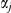 equals the prior mean 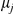. If 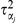 = ∞, 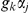 is actually estimated using least squares and the parameters 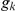 and 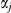 cannot be distinguished. If 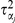 is finite, the coefficient 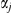 is shrunk towards but not identical to the prior mean 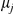. Therefore, the prior distributions bridge the gap between the two extremes of simply using the fixed weighted sum 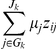 of the *J_k_* variants as a predictor (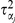 = 0), and including them as *J_k_* independent predictors (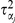 = ∞) [37], [38]. This interpretation can be more explicitly understood by the identity
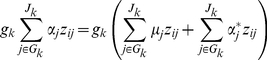
(6)where 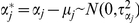. The second term in the right side is controlled by the variances 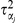, and represents the deviation from the fixed weighted sum 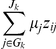. Most of existing methods for analyzing rare variants proceed to construct a linear combination (genetic score) of rare variants with fixed weights [21]–[25], and thus can be viewed as special cases of our model.

The prior means 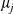 represent the prior relative importance of the individual variants and can be specified in various ways. The weights proposed by previous methods [21]–[25] can be used as the prior means 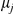 in our hierarchical model. The simplest way is to set all 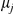 = 1, resulting in the simple sum 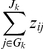, and incorporating no prior information about the relative importance of rare variants into the model. But our method can estimate the weights from data and produce different weights to different variants based on their contributions to the phenotype. Therefore, our model uses a previous score (i.e., 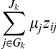) as the baseline, and improves the fit by accounting for the variation among individual variants.

An alternative choice of the prior means is to set all 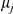 = 0. With this choice, the weights are shrunk towards zero, and variants with small effects can be essentially removed from the model. This seems to be reasonable, especially for the situations with non-functional variants. However, we don't recommend this approach for rare variants for several reasons. First, most of rare and common variants have small effects, but they can be cumulatively important. In order to detect the cumulative effect, therefore, it would be better to include all the small effects in the model. Second, the estimated group effect can be less interpretable and accurate, if only one or a few variants are included in the model. Third, our hierarchical model can estimate the weights of individual variants from the data, and thus can deal with non-functional variants and disparate effects.

### Model Fitting and Inference

Our Bayesian hierarchical GLMs can be fitted using Markov chain Monte Carlo (MCMC) algorithms that fully explore the joint posterior distribution by alternately sampling each parameter from its conditional posterior distribution [36]. However, it is desirable to have a faster computation that provides a point estimate (i.e., the posterior mode) of the coefficients and their standard errors (and thus the *p*-values). Such an approximate calculation has been routinely applied in statistical practice [41]. We develop our mode-finding algorithm by modifying the standard iterative weighted least squares (IWLS) for fitting classical generalized linear models [42], [43].

Our algorithm updates the coefficients 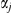 and 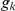 using an iterative procedure. Conditional on the current estimates 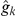, we update 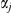 by running the generalized linear model with the proposed prior distributions for 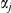 and other corresponding parameters:
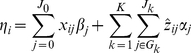
(7)where 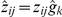, and then conditional on the current estimates 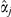, we update 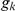 by running the generalized linear model with the proposed prior distributions for 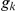 and other corresponding parameters:
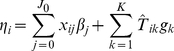
(8)where 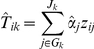. We fit these two hierarchical generalized linear models by incorporating a flexible expectation-maximization (EM) algorithm into the iteratively weighted least squares (IWLS) for fitting classical generalized linear models. We describe our EM-IWLS algorithm in detail in Text S1.

We initialize our iterative algorithm by setting the parameters (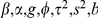) with some plausible values. For example, we can start with 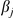 = 0, 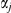 = 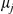, 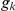 = 1, 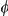 = 1, 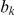 = 0.5, 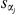 = 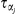 = 0.5, and 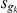 = 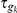 = 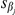 = 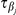 = 1. We then update the parameters by iteratively running the hierarchical generalized linear models (7) and (8) until convergence. Instead of doing a nested converged EM-IWLS for each of the two models, we can run one step of the EM-IWLS at each iteration, thus taking less computing time to ultimately achieve convergence by not wasting time running many steps of the EM-IWLS within each iteration. To assess convergence, we use the standard criterion for analysis of classical generalized linear models (as implemented in the R function glm), i.e., 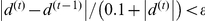, where 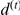 is the estimate of deviance (i.e., 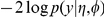) at the *t* ^th^ iteration, and 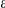 is a small value (say 10^−5^).

At convergence of the algorithm, we summarize the inferences using the latest estimates of the coefficients 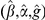 and their standard errors. Based on these outputs, we can calculate approximate *p*-values as in the classical framework for testing whether a coefficient is significantly different from zero, for example, the hypothesis 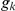 = 0. The adjusted main effects 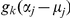 are then estimated as 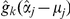, and the approximate standard error for 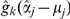 can be obtained by using the delta technique:
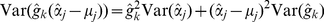
(9)Therefore, we can calculate the approximate *p*-value for testing the hypothesis 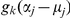 = 0.

### Implementation

Our model fitting strategy is based on extending the well-developed IWLS algorithm for fitting classical GLMs to our Bayesian hierarchical GLMs. The IWLS algorithm is executed in the glm function in R (http://www.r-project.org/). We have implemented the EM-IWLS algorithm by inserting the E-step for updating the missing values (i.e., the variances 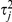 and the hyperparameters 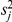 and 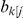) and the steps for calculating the augmented data and the dispersion parameter into the IWLS procedure (see Text S1). We have created a new R function bglm by modifying the glm function to implement our EM-IWLS algorithm that estimates posterior modes and standard deviations for hierarchical GLMs with the prior distributions proposed here (see Text S1) and some other hierarchical priors [44], [45]. We have also developed an R function bglm.ex that implements the iterative algorithm described above for fitting our hierarchical multiplicative GLMs. Although described in the context of genetic variants in this paper, the functions bglm and bglm.ex can be used as general tools for routine data analysis using hierarchical GLMs. We have incorporated the functions bglm and bglm.ex into the freely available R package BhGLM (http://www.ssg.uab.edu/bhglm/) that is an extensible and interactive environment for genetic association analysis of common and rare variants and gene-gene and gene-environment interactions.

### Alternative Approaches

Our hierarchical multiplicative GLMs include various models as special cases. Although less comprehensive, these reduced models can be useful in some situations, and thus can be used as alternative approaches to analysis of multiple groups of rare and common variants. We here consider two types of reduced models. The first ignores the group effects and directly models the main effects of individual variants. Thus, the linear predictor (1) is reduced to
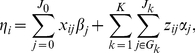
(10)and the mean and the distribution of the response variable take the same form of the expressions (2) and (3). In this model, the coefficient 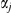 represents the main effect of genetic variant *j*, and follows the hierarchical prior distribution (4) with the prior mean 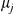 = 0. This approach can only detect individual variants with strong effects, and is less powerful in situations where the effects of all individual variants are small but they are cumulatively significant.

The second alternative approach is to preset the weights of individual variants using the previous methods [21]–[25]. Thus, the linear predictor (1) becomes
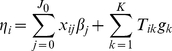
(11)where 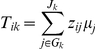 with fixed weights 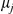. This model is equivalent to setting the priors as 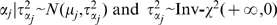 (i.e., 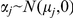) and thus is a special case of our hierarchical model. The performance of this method heavily depends on the quality of the fixed weights.

## Results

### Application: Population-Based Resequencing of *ANGPTL4* and Triglycerides

#### Description of dataset

**Figure 1:**
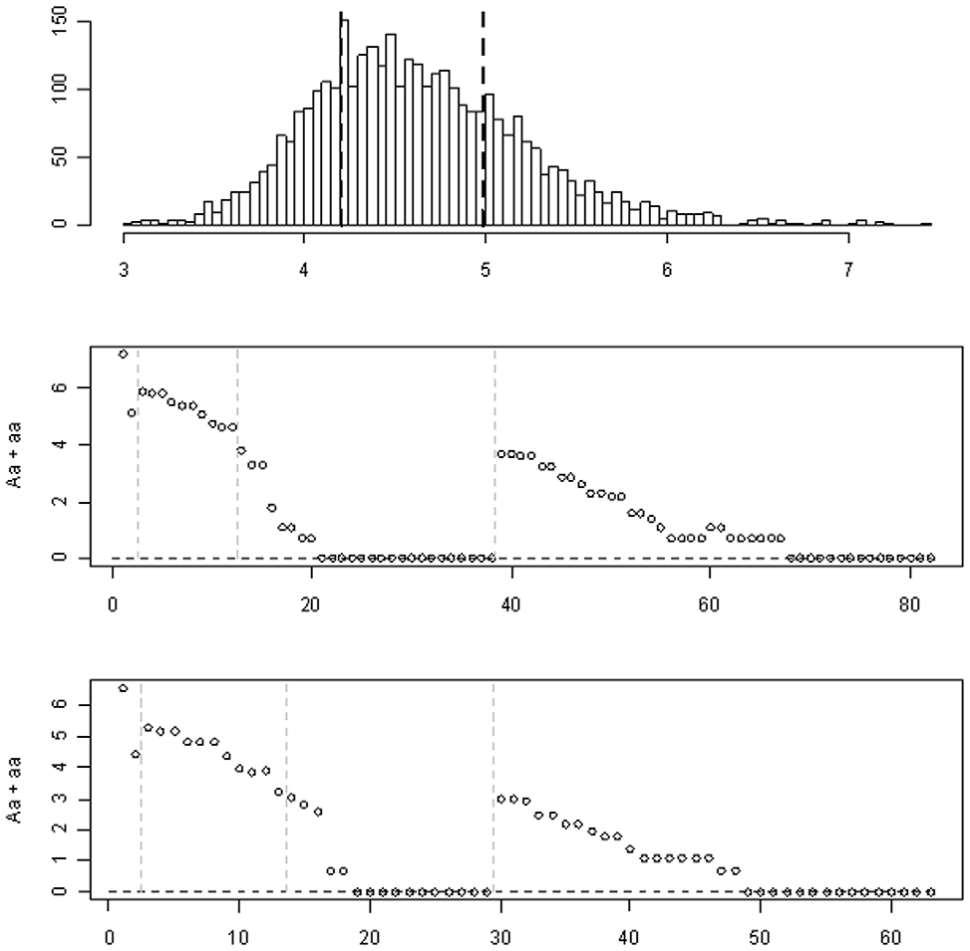
The Dallas Heart Study data set. The top panel: the histogram of the log-transformed plasma levels of triglyceride and the 25th and 75th percentiles (the black dotted lines). The middle panel: the logarithm of the observed count of heterozygotes (Aa) and rare homozygotes (aa) for each variant in the continuous trait analysis. The bottom panel: the logarithm of the observed count of Aa and aa for each variant in the binary trait analysis. The gray dotted lines show the four groups: common non-synonymous, common synonymous, rare non-synonymous, and rare synonymous.

Romeo et al. [13] was the first application of resequencing to a large population to examine the role of the adipokine gene *ANGPTL4* in lipid metabolism. The study included the 3,551 participants of the Dallas Heart Study (DHS) from whom fasting venous blood samples were obtained. The DHS is a population-based random sample of Dallas County residents, consisting of 601 Hispanic (H), 1,830 African American (AA), 1,045 European American (EA) and 75 other ethnicities. The 75 participants from other ethnicities will be excluded from our analysis. The phenotype analyzed in our study is the log-transformed plasma levels of triglyceride. The top panel of Figure 1 shows the histogram of this continuous phenotype and the 25th and 75th percentiles. Following the analysis of Romeo et al. [13], we also considered a binary trait, coding individuals in the bottom and top quartiles of the distribution as 0 and 1, respectively, and excluding other individuals from the analysis. Hereafter, we refer these two phenotypes as the *continuous* and *binary* traits. Our analyses included race (a three-level factor), age, sex, and BMI as covariates in the model. We excluded individuals with any missing values of the covariates from the analysis, resulting in 3008 and 1499 individuals in the analyses of the continuous and binary traits, respectively.

Romeo et al. [13] sequenced the seven exons and the intron-exon boundaries of the gene *ANGPTL4*, and identified a total of 93 sequence variations. After removing variants that were not segregating in the sample, the numbers of variants reduced to 82 and 63 for the analyses of the continuous and binary traits, respectively. Most of these variants were rare: only 12 and 13 variants had a minor allele frequency above 1%, and 33 and 26 variants were found only in one object in the two analyses, respectively (see Figure 1).

#### The methods

We divided the variants into four groups: common non-synonymous, common synonymous, rare non-synonymous, and rare synonymous. We used a minor allele frequency of 1% as the threshold to distinguish between common and rare variants [21]. Figure 1 displays the four groups of variants and the logarithm of the observed count of heterozygotes (Aa) and rare homozygotes (aa) for each variant. The four groups consisted of 2 (2), 10 (11), 26 (16) and 44 (34) variants for the analyses of the continuous (binary) traits, respectively. Since there are only two common non-synonymous variants (i.e., 8155_T266M and 8191_R278Q), we did not estimate their group effect and instead treated them as two covariates in the models. We coded the main-effect predictor of each variant using the additive genetic model, i.e., the number of minor alleles in the observed genotype. The genotypes of the variants contained ∼3%–16% missing values. For the missing genotypes, we filled in the variables using the expectation of the observed values in that marker. This simple, but reasonable, imputation method is computationally much more efficient than comprehensive methods using MCMC algorithms or multiple imputations and has been widely used in genetic association studies. The previous studies and the analyses in this work show that this imputation method yields a reasonable result [43].

We first analyzed the data using the hierarchical multiplicative GLMs (Equations 1–3) with the proposed hierarchical prior distributions (Equations 4 and 5). For comparisons, we then used three alternative methods: 1) Setting all the scale parameters 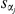 in the hierarchical prior (4) to a known value (e.g., 0.5). This is an extension of Yi and Zhi [26] to multiple groups of variants; 2) Setting the weights of individual variants to fixed values 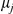 (see Equation 11). This simply extends the previous Simple-Sum [22], [27] and Weighted-Sum methods [24]; 3) Ignoring the group effects and directly estimating the main effects of all individual variants (see Equation 10).

All the analyses simultaneously fitted all the non-genetic variables (i.e., race, age, sex, and BMI), the two common non-synonymous variants (i.e., 8155_T266M and 8191_R278Q) and the three groups of variants. We used a normal regression and a logistic regression for the continuous and the binary traits, respectively. We set the prior means 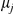 in two ways; the first is to set 1 for all the variants, and the second is to set 1 for the synonymous variants and the predicted functional scores for the rare non-synonymous variants. The functional scores were calculated using the software PolyPhen [24], [46]. The iterative EM-IWLS algorithm started from the plausible initial values described earlier and took 12 (16) iterations to reach convergence for the analysis of the continuous (binary) trait (∼0.1 minutes on a P4 desktop computer).

#### Results of data analyses

**Figure 2:**
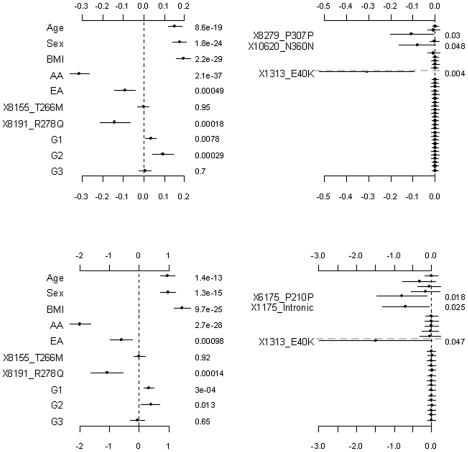
Analyses of the proposed hierarchical GLMs with prior means *μ_j_* = 1 for all variants. The top and bottom panels are for the continuous and binary traits, respectively. The left panel is for the covariates, the two common non-synonymous variants and the three group effects (G1: common synonymous; G2: rare non-synonymous; G3: rare synonymous). The right panel is for the adjusted main effects (the gray dotted line shows the two groups G1 and G2). The points, short lines and numbers at the right side represent estimates of effects, ±2 standard errors, and *p*-values, respectively. Only adjusted main effects with *p*-value <0.1 are labeled.

**Figure 3:**
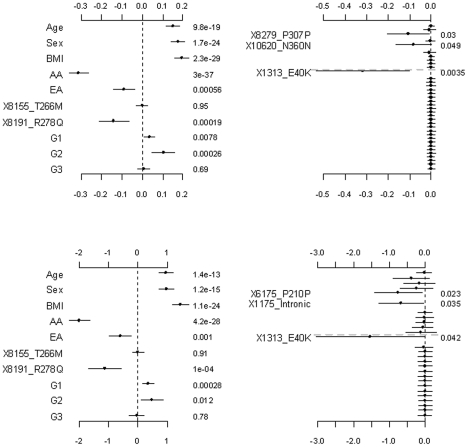
Analyses of the proposed hierarchical GLMs with prior means *μ_j_* being the functional probabilities for the rare non-synonymous variants. The top and bottom panels are for the continuous and binary traits, respectively. The left panel is for the covariates, the two common non-synonymous variants and the three group effects (G1: common synonymous; G2: rare non-synonymous; G3: rare synonymous). The right panel is for the adjusted main effects (the gray dotted line shows the two groups G1 and G2). The points, short lines and numbers at the right side represent estimates of effects, ±2 standard errors, and *p*-values, respectively. Only adjusted main effects with *p*-value <0.1 are labeled.

**Figure 4:**
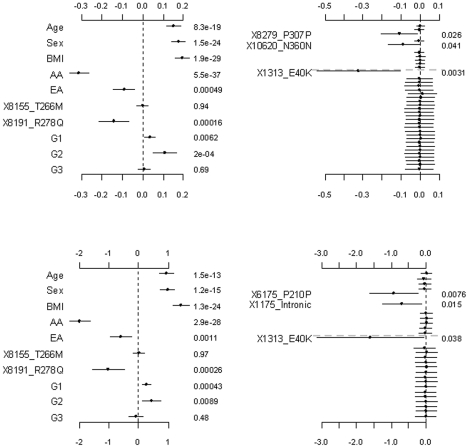
Analyses of the hierarchical GLMs with fixed scale 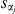 = 0.5. The top and bottom panels are for the continuous and binary traits, respectively. The left panel is for the covariates, the two common non-synonymous variants and the three group effects (G1: common synonymous; G2: rare non-synonymous; G3: rare synonymous). The right panel is for the adjusted main effects (the gray dotted line shows the two groups G1 and G2). The points, short lines and numbers at the right side represent estimates of effects, ±2 standard errors, and *p*-values, respectively. Only adjusted main effects with *p*-value <0.1 are labeled.

**Figure 5:**
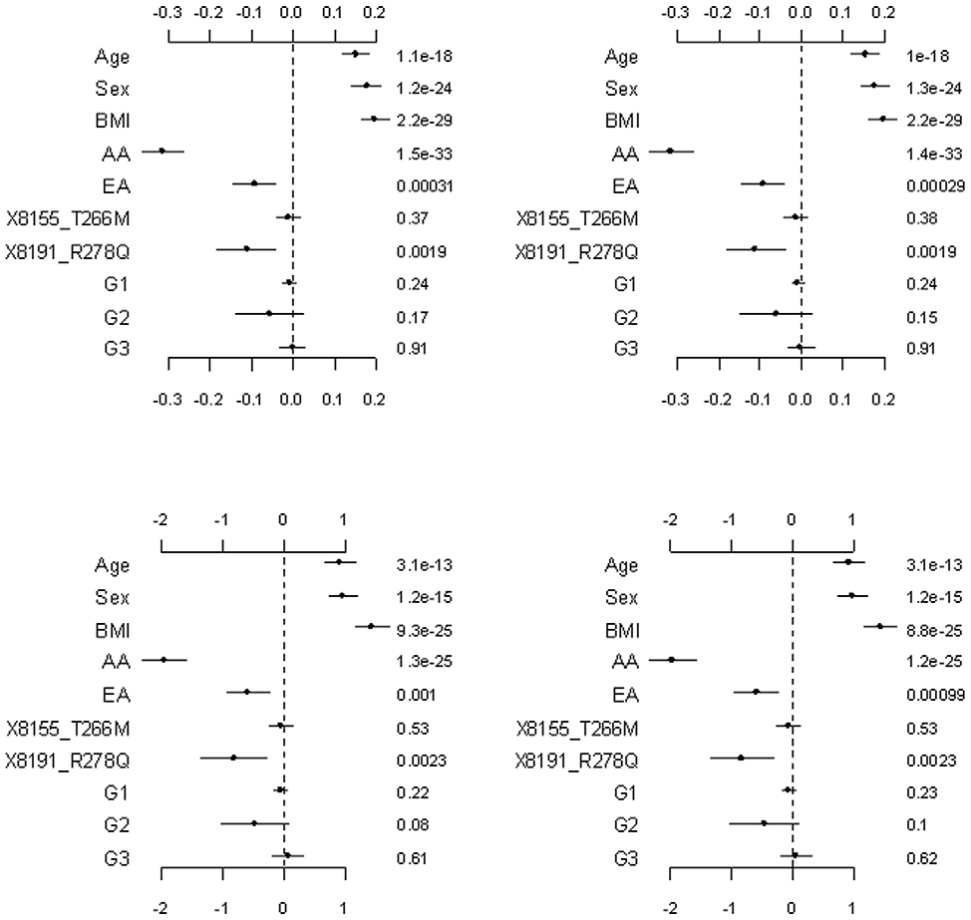
Setting the weights of individual variants to fixed values 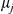. The top and bottom panels are for the continuous and binary traits, and the left and right panels are for Simple-Sum and Weighted-Sum methods, respectively. The points, short lines and numbers at the right side represent estimates of effects, ±2 standard errors, and *p*-values, respectively.

**Figure 6:**
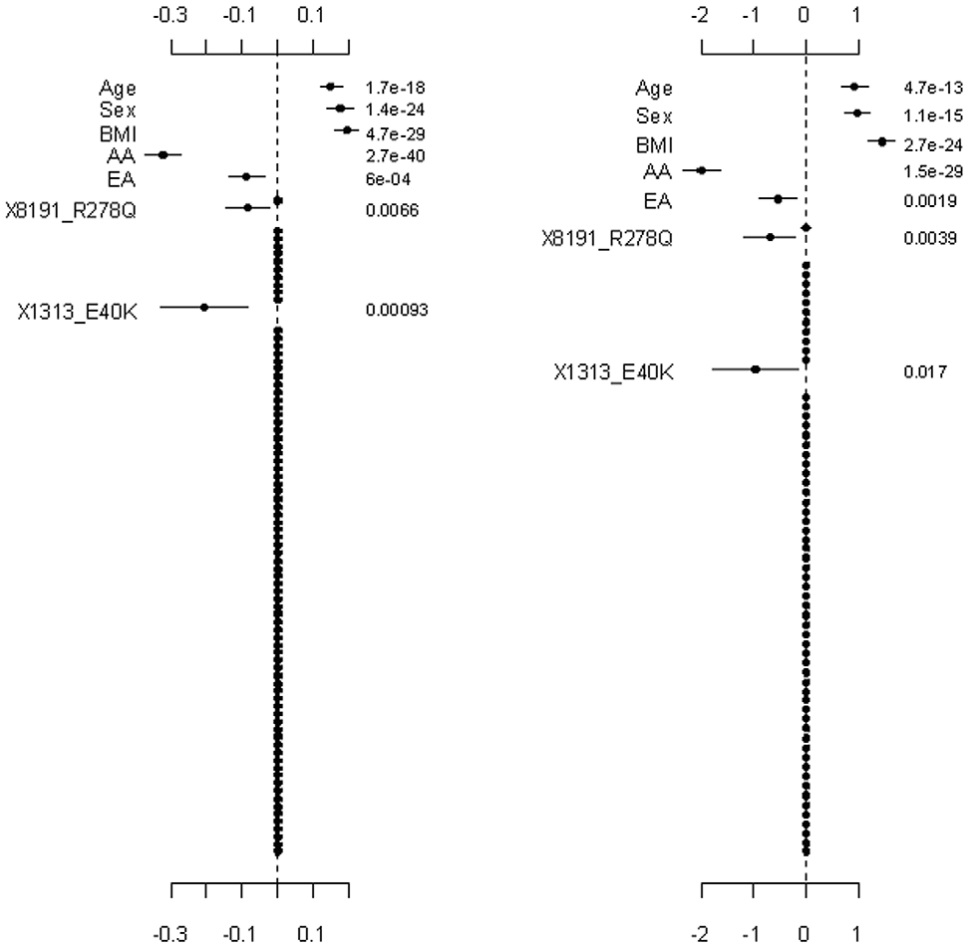
Ignoring the group effects and directly estimating the main effects of individual variants. The left and right panels are for the continuous and binary traits, respectively. The points, short lines and numbers at the right side represent estimates of effects, ±2 standard errors, and *p*-values, respectively. Only main effects with *p*-value <0.1 are labeled.

Figure 2 shows the results from the analyses of the proposed hierarchical GLMs with prior means *μ_j_* set to 1 for all the grouped variants. All the non-genetic covariates and the common non-synonymous variant 8191_R278Q were found to significantly affect both the continuous and the binary traits. These effects were also significant under the other models considered (see Figure 3, Figure 4 Figure 5, Figure 6). Although not mentioned in Romeo et al. [13], our analyses detected the minor allele of the variant 8191_R278Q to significantly decrease triglyceride levels, consistent with the finding of King et al. [25]. In addition to the significant non-genetic and genetic covariates, we identified two significant group effects, i.e., those of the common synonymous and the rare non-synonymous variants. These two group effects remained significant even corrected for multiple testing using the method of Benjamini and Hochberg [47]. Our results were fairly consistent with the original analyses; Romeo et al. [13] observed that the number of individuals with nonsynonymous variants in the bottom quartile was significantly greater than the number in the highest quartile, but the number of synonymous variants in the upper and lower tails of the distribution was identical. Since we divided synonymous variants into two groups, our analyses produced additional findings; the group effect of the common synonymous variants was significant, but that of the rare synonymous variants was insignificant.

The group effects in our model should be interpreted with caution; a positive group effect does not necessarily mean that the variants increase the phenotype, because for some variants the weights can be estimated to be negative (for example, the rare variant 1313_E40K in our analyses). The right panel of Figure 2 displays the adjusted main effects of the common synonymous and the rare non-synonymous variants, thus showing which variants are more important. Our analyses identified the rare variant 1313_E40K as the most important. The negative adjusted main effect indicated that the minor allele of 1313_E40K decreases triglyceride levels. Romeo et al. [13] and King et al. [25] also found that the variant 1313_E40K significantly decreased triglyceride levels. Therefore, the proposed method can simultaneously identify significant group effects and individual variants.

Figure 3 shows the results for the proposed hierarchical GLMs with prior means *μ_j_* set to the functional probabilities for the rare non-synonymous variants, which were estimated to range from 0.16 to 1. These models produced qualitatively identical results as the above analyses, but slightly lower p-values for the significant effects. Price et al. [24] showed that incorporating computational predictions of functional importance can increase power for pooled association tests for rare variants.

Figure 4, Figure 5, and Figure 6 display the results from the three alternative approaches. The models setting all the scale parameters 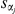 in the hierarchical prior (Equation 4) to 0.5 (as suggested by Yi and Zhi [26]) produced results similar to the previous analyses (Figure 4). However, this alternative method inflated the standard deviations for the estimates of the weights. The second alternative method preset the weights to 1 for all the variants or the estimated functional probabilities for the rare non-synonymous variants. But, these simpler models did not detect any significant group effects (Figure 5). Our third alternative approach simultaneously fitted the main effects of all the covariates and common and rare variants. As expected, this hierarchical model was able to detect only large effects. The variants 8191_R278Q and 1313_E40K were found to have strong effects in our previous analyses and thus were also detected in this alternative analysis.

### Simulation Studies

#### Simulation design

We used simulations to validate the proposed models and algorithm and to study the properties of the method. Although most published simulation studies of rare variants generated genotypes assuming a population genetics model for the propagation of rare variants, the best way will be to take real sequence data obtained from many individuals and simulate phenotypes based on variants in those sequences, making assumptions only about genetic effects of variants [19]. Thus, we performed simulation studies by taking advantage of the real genotypes of common and rare variants and also the covariates in the above large real dataset.

We evaluated some factors that may affect the performance of the methods:

- *Sample size:* We considered two sample sizes, including all observations in the real data (n = 3008) or individuals in the bottom and top quartiles of the real continuous phenotype (n = 1499), respectively.
- *Number of groups and number of variants in each group:* We first considered the three groups of variants (i.e., common synonymous, rare non-synonymous, and rare synonymous) as in our real analyses. We then considered the second scenario with six groups by randomly partitioning each group into two with equal number of variants.
- *Genetic effect sizes and directions of variants:* For each group of variants, we first assumed the total heritability (*h*) explained by the variants and the proportion of negative additive effects (*p.neg*) for the variants. We then randomly sampled an additive effect *β_j_* for each variant from the region [0, *β_h_*] and changed the sign of *β_j_* with the probability *p.neg*. The upper bound *β_h_* was calculated using the method of Yi and Zhi [26], which controlled the total heritability of each group of variants approximately equal to the assumed value *h*. We considered several combinations of different *h* and *p.neg* (see Tables 1 and 2). The assumed total heritabilities were approximately equal to the estimated values of significant groups in our real data analyses.

**Table 1:** Simulations with three groups.

Group		1	2	3
Number of variants		10 (11)	26 (16)	44 (34)
Scenario	a	*h* = 0%	*h* = 0.5%*p.neg* = 0	*h* = 0%
	b	*h* = 0%	*h* = 0.7%*p.neg* = 0.4	*h* = 0%
	c	*h* = 0.5%*p.neg* = 0	*h* = 0.5%*p.neg* = 0	*h* = 0%
	d	*h* = 0.7%*p.neg* = 0.4	*h* = 0.7%*p.neg* = 0.4	*h* = 0%
	e	*h* = 0%	*h* = 0.7%*p.neg* = 0	*h* = 0.7%*p.neg* = 0
	f	*h* = 0%	*h* = 0.7%*p.neg* = 0.4	*h* = 0.7%*p.neg* = 0.4

The number of variants in each group for simulations with n = 3,008 (1,449), the assumed total heritability (*h*) explained by each group of variants, and the proportion of negative additive effects (*p.neg*) for groups with non-zero *h*.

**Table 2:** Simulations with six groups.

Group		1	2	3	4	5	6
Number of variants		5 (5)	5 (6)	13 (8)	13 (8)	22 (17)	22 (17)
Scenario	a	*h* = 0%	*h* = 0.5%*p.neg* = 0	*h* = 0%	*h* = 0%	*h* = 0.5%*p.neg* = 0	*h* = 0%
	b	*h* = 0%	*h* = 0.7%*p.neg* = 0.4	*h* = 0%	*h* = 0%	*h* = 0.7%*p.neg* = 0.4	*h* = 0%
	c	*h* = 0%	*h* = 0%	*h* = 0.5%*p.neg* = 0	*h* = 0%	*h* = 0.5%*p.neg* = 0	*h* = 0%
	d	*h* = 0%	*h* = 0%	*h* = 0.7%*p.neg* = 0.4	*h* = 0%	*h* = 0.7%*p.neg* = 0.4	*h* = 0%

The number of variants in each group for simulations with n = 3,008 (1,449), the assumed total heritability (*h*) explained by each group of variants, and the proportion of negative additive effects (*p.neg*) for groups with non-zero *h*.

We simulated a continuous and a binary phenotype. As in our real data analyses, we simultaneously fitted all the non-genetic variables (i.e., race, age, sex, and BMI), the two common non-synonymous variants (i.e., 8155_T266M and 8191_R278Q) and the grouped variants. We assumed the non-genetic coefficients and the additive effects of 8155_T266M and 8191_R278Q to be their estimated values in the continuous trait analysis (see the top panel of Figure 2). Given the assumed and the simulated coefficients, we first generated a normal continuous trait by setting the residual standard deviation to the estimated value in the continuous trait analysis (≈0.2), and then set half of individuals with the 50% largest continuous phenotype as ‘affected’ (*y_i_* = 1) and the other individuals as ‘unaffected’ (*y_i_* = 0) to create a binary trait [26].

For each situation, 1000 replicated datasets were simulated. We calculated the frequencies of each effect estimated as significant at the threshold levels of *α* = 0.05, 0.01, and 0.001 over 1000 replicates. These frequencies corresponded to the empirical power for the simulated non-zero effects and the type I error rate for other coefficients, respectively. We compared the proposed method with the three alternative approaches described in the above real data analyses. For the proposed method and the alternative methods with fixed scale parameters or fixed weights, we can calculate powers or type I error rates for all the covariates and the group effects. Since the third alternative approach cannot estimate the group effects, we simply used the minimal p-value to calculate powers or type I error rates for each group of variants. For each situation, the iterative EM-IWLS algorithm started from the plausible initial values described earlier and ran until convergence.

#### Results of simulations

**Figure 7:**
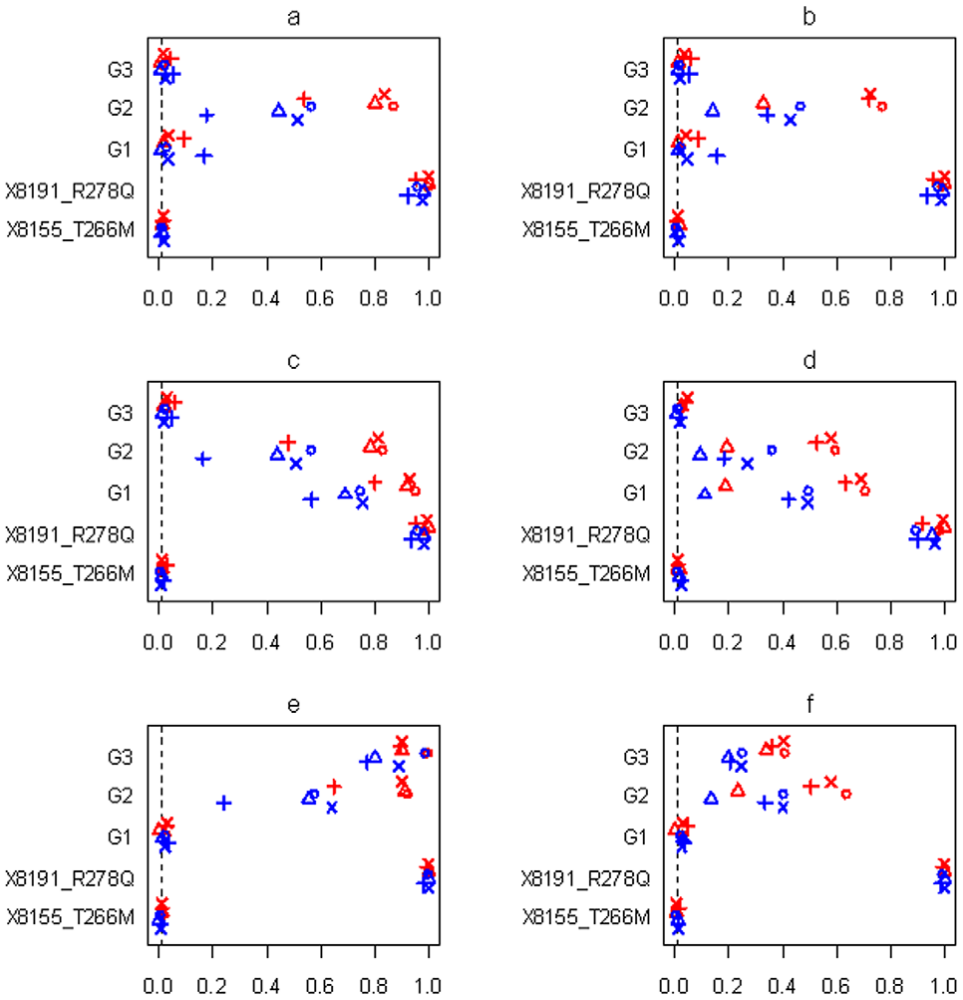
Simulations with sample size n = 3,008 and three groups for the six scenarios (see **Table 1**). Power or Type I error rate for the proposed method (○), Yi and Zhi (×), Simple-Sum (Δ) and All-Variants (+) under the threshold level of 0.01. X8155_T266M and X8191_R278Q are the two common non-synonymous variants, and G1, G2 and G3 are the three group effects (G1: common synonymous; G2: rare non-synonymous; G3: rare synonymous). Red and blue symbols represent results for continuous and binary traits, respectively. The dashed line is the nominal 0.01 level.

**Figure 8:**
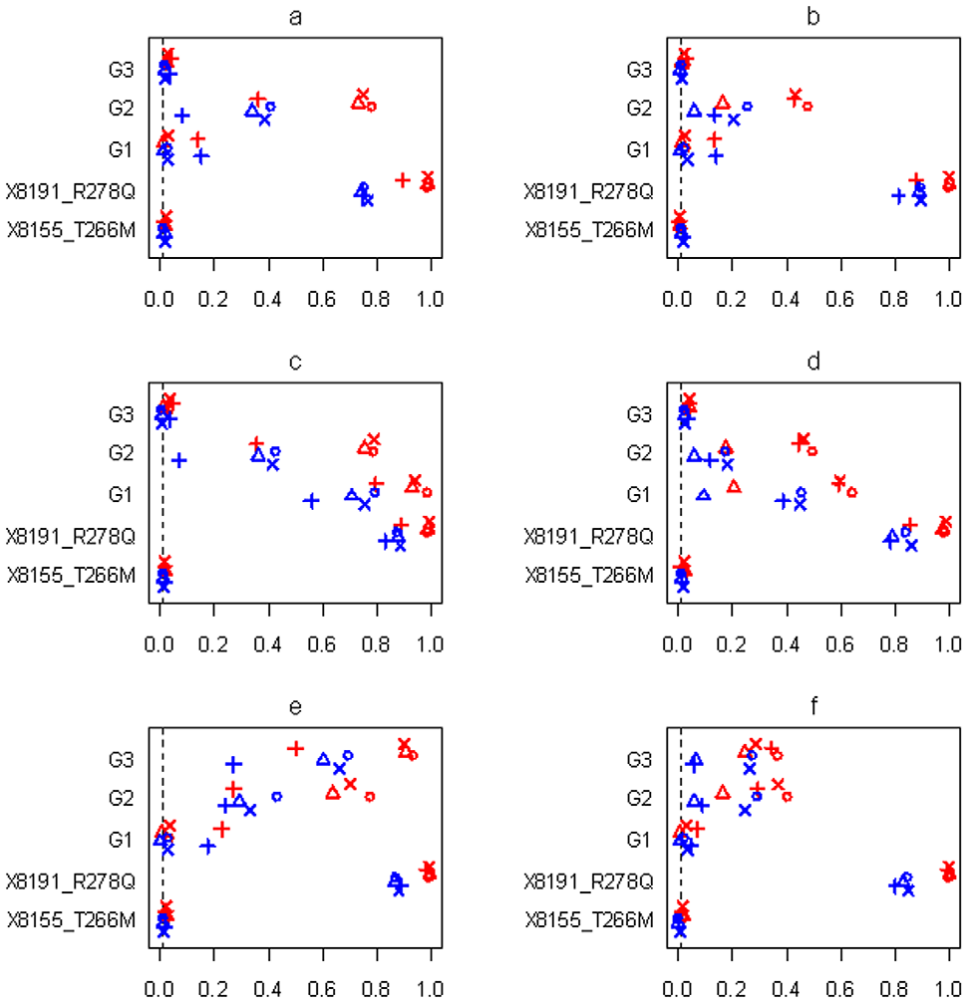
Simulations with sample size n = 1,499 and three groups for the six scenarios (see **Table 1**). Power or Type I error rate for the proposed method (○), Yi and Zhi (×), Simple-Sum (Δ) and All-Variants (+) under the threshold level of 0.01. X8155_T266M and X8191_R278Q are the two common non-synonymous variants, and G1, G2 and G3 are the three group effects (G1: common synonymous; G2: rare non-synonymous; G3: rare synonymous). Red and blue symbols represent results for continuous and binary traits, respectively. The dashed line is the nominal 0.01 level.

**Figure 9:**
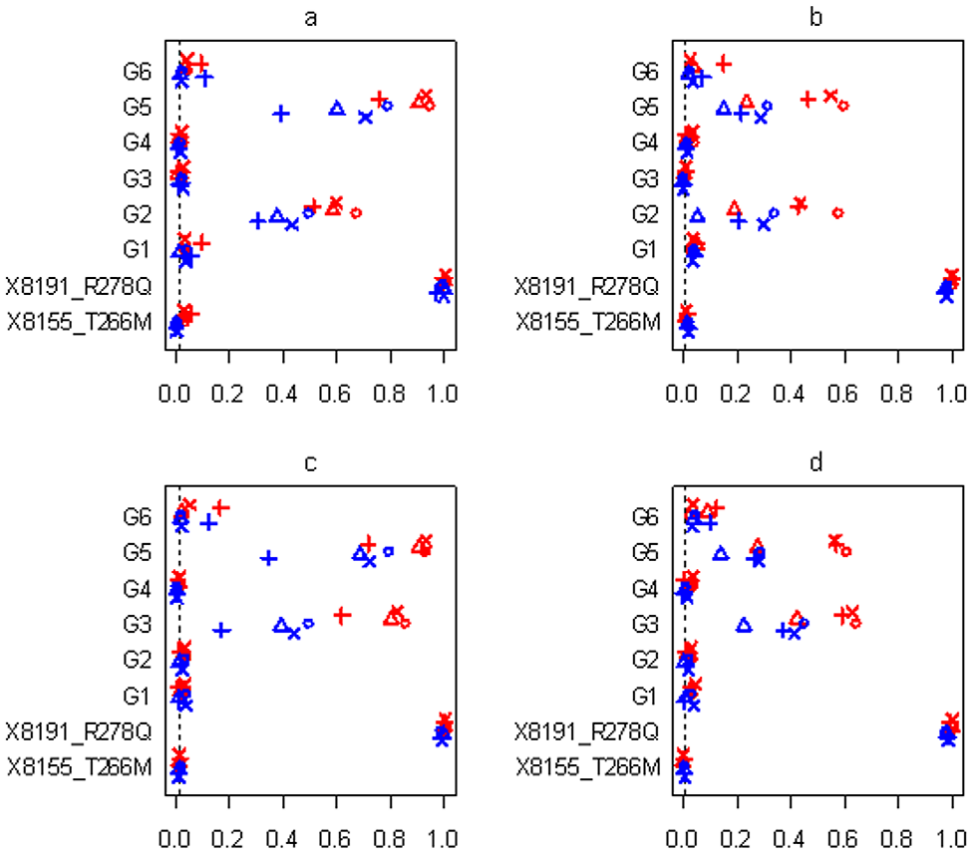
Simulations with sample size n = 3,008 and six groups for the four scenarios (see **Table 2**). Power or Type I error rate for the proposed method (○), Yi and Zhi (×), Simple-Sum (Δ) and All-Variants (+) under the threshold level of 0.01. X8155_T266M and X8191_R278Q are the two common non-synonymous variants, and G1–G6 are the six group effects. Red and blue symbols represent results for continuous and binary traits, respectively. The dashed line is the nominal 0.01 level.

Figure 7 and Figure 8 display the results at the threshold level of 0.01 from simulations with three groups and sample sizes of 3008 and 1449, respectively. Figure 9 shows the results from simulations with six groups and sample size of 3008. In all the simulations, the non-genetic covariates (race, age, sex, and BMI), which were highly significant in the real data analyses, were detected with high power by all the methods (not shown in the figures). All the methods also had high power to detect the significant common variant 8191_R278Q, and low type I error for the insignificant common variant 8155_T266M, showing that the genetic effects of common variants can be effectively estimated in large-scale studies.

In all the simulation scenarios, the proposed method and the extension of Yi and Zhi [26] were consistently more powerful to detect the simulated group(s) of variants than the other methods. As expected, the power drastically increases with larger sample size and for continuous phenotype. These relationships hold rather generally for the methods that we examined. In the simulations with three groups, the group of common variants was detected with slightly higher power than the group of rare variants (see Figure 7c and 7d, Figure 8c and 8d). However, when the number of common variants was too small, their group effect was detected with lower power than the group of rare variants (see Figure 9a). Our simulations showed that the proposed method and the extension of Yi and Zhi [26] also well controlled the type I error for groups with zero effects. This results from the fact that our hierarchical priors can shrink the weights of zero-effect variants to the prior means and thus yield the genetic score approximately equal to the simple sum or weighted sum. Our simulations also showed that the method ignoring group effects had the highest type I error rate.

For the groups in which all variants affected the traits in the same direction, the summation of the additive-effect predictors could provide a useful genetic score of these variants, and thus the simple-sum method had reasonable power to detect the group effect (see Figure 7a, 7c, 7e; Figure 8a, 8c, 8e; Figure 9a, 9c). Even in this situation, however, the proposed method and the extension of Yi and Zhi (2011) [26] were still more powerful than the simple-sum method. This may result from the fact that these hierarchical models estimate the weights from data and thus can produce different weights for different variants based on their contributions to the phenotype. The simulations further showed that the proposed method had slightly higher power than Yi and Zhi (2011) [26]. This is likely the results that the proposed method introduces variable-specific shrinkage parameters and thus could estimate the weights of variants more effectively. Finally, we found that the method ignoring group effects had the lowest power. This is expected because with the low total heritability the effects of single variants were very small and could not be detected powerfully.

For the groups in which 60% of variants increase disease risk and others are disease-protective, the summation of the additive-effect predictors provides an inefficient genetic score to summarize the information of the variants, and thus the simple-sum method had low power to detect the association (see Figure 7b, 7d, 7f; Figure 8b, 8d, 8f; and Figure 9b, 9d). These results are expected because using equal weights for disease-causing and disease-protective variants the information across variants can be cancelled out and the true association cannot be detected. However, the proposed method and the extension of Yi and Zhi (2011) [26] still had reasonable power to detect these multiple rare and common variants with opposite effects. These hierarchical models could yield opposite weights for disease-causing and protective variants, and thus avoid cancellation of individual-variant variation. Once again, we found that the proposed method had slightly higher power than Yi and Zhi (2011) [26]. Interestingly, our simulations show that the hierarchical models of all variants were not influenced by opposite effects, because they directly estimate the effects of individual variants; for a higher total heritability of multiple variants, some variants had larger effects and thus could be detectable individually.

We also evaluated power and type I error at several different levels (e.g., *α* = 0.05, 0.001). The conclusions described above generally held (data not shown). Yi and Zhi [26] found that the hierarchical GLMs uniformly yielded much lower *p*-values for the simulated group effects than the previous methods. This was also true for the proposed method in our simulation studies. This indicates that our method usually provides stronger evidence of association if the variants really influence the disease.

## Discussion

We have proposed here a Bayesian hierarchical generalized linear model framework for simultaneously analyzing multiple groups of rare and common variants and relevant covariates. Since complex diseases and traits are likely influenced by multiple genetic variants and environmental factors, the joint analyses of multiple groups of genetic variants can improve the power of detecting causal effects and lead to increased understanding about the genetic architecture of diseases. The proposed hierarchical generalized linear models introduce a group effect and a genetic score for each group of variants, and jointly estimate the group effects and the weights of the genetic scores. This can produce ‘optimal’ weights to different variants based on their contributions to the phenotype, yielding an effective summary of the information across variants. The simulation studies show that the proposed method can consistently provide reasonable power even in the presence of both risk and protective variants in a group, and has better power than existing approaches even when all variants act in the same direction. Application of the method to a large published dataset on resequencing of the gene *ANGPTL4* and triglycerides not only confirmed the original findings but also detected new associations.

In addition to the properties described above, our method has several remarkable features. First, the proposed method can simultaneously estimate the group effects of multiple groups of variants and the individual effects of the variants, allowing us to not only identify significant genes (or groups of variants) but also assess the relative importance of single variants. Second, our hierarchical model includes various existing methods for rare variants as special cases. This shows that the proposed method is theoretically more advantageous than the existing methods, and allows us to conveniently analyze data using different ways. Third, any external information about variants, for example, the functional prediction, can be easily incorporated into our hierarchical model by specifying the prior means of the weights for variants. By doing so, our approach has the additional advantage of accounting for uncertainties about the prior assumptions. Fourth, our approach is based on the generalized linear model framework and thus can deal with various types of continuous and discrete phenotypes and covariates, and can fit any generalized linear models. Finally, the proposed algorithm extends the standard procedure for fitting classical generalized linear models in the general statistical package R to our Bayesian model, leading to the development of stable and flexible software.

Our approach is highly extensible; we have planned several extensions to the proposed method, some of which have been initially implemented in our software BhGLM. The key to our approach is the use of hierarchical prior distributions for the weights and the group effects, so that these multiplicative parameters are identifiable and can be simultaneously estimated from the data. We have proposed to use the hierarchical expression of the half-Cauchy distribution with the innovation of introducing both group- and variable-specific parameters. The half-Cauchy prior is an excellent default choice for many problems [39], [40], and has been shown to perform well for our purposes. However, other hierarchical priors or penalized likelihood methods have been developed for high-dimensional data analysis, including lasso [48], [49], adaptive Lasso [50], and the elastic net [51]. These methods can be expressed as hierarchical models by assigning certain priors on the variances and other hyperparameters [45], [48], [52], and can be incorporated into our framework.

Although demonstrated with only several groups of variants, our method can be adapted to deal with large-scale sequencing data involving thousands of exomes or candidate genes. For these high-dimensional settings, we need to modify the prior distributions of the group effects and the computational algorithm. We can place a shrinkage prior on the inverse scale in the gamma prior of 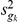 and estimate the inverse scale from the data. We can further group the group effects based on pathways that candidate genes belong to, and specify the shrinkage priors by incorporating the second-level hierarchical structure, similar to the hierarchical priors of the weights. We describe our algorithm by simultaneously estimating all weights. This method can be very fast when the number of variables is not very large (say <2000) and has the advantage of accommodating the correlations among all the variables. However, it can be slow or even cannot be implemented when the number of variables is large due to memory storage and convergence problems. We can extend the algorithm to update coefficients group by group; at each of the iteration, the group-at-time algorithm proceeds by cycling through all the groups of parameters and treats the linear predictor of all other groups as an *offset* in the model. This method updates coefficients in a conditional manner, significantly reducing the number of parameters in each M-step of the EM-IWLS algorithm, and thus can deal with large number of variables.

Our third extension could incorporate external gene or pathway level information into the hierarchical model. Candidate genes or pathways studies usually consist of data at different levels, i.e., genetic variants within multiple candidate genes or pathways which may be functionally related [53]. Most of statistical methods for association studies consider only individual-level predictors (i.e., SNPs and covariates) and ignore the hierarchical structure of the data and gene or pathway-level information. Often, rich gene or pathway-level information is available [54], including genomic annotation or pathway ontologies, functional assays, *in silico* predictions of function or evolutionary conservation [55]. Therefore, there is a growing need to develop sophisticated approaches that model the multilevel variation simultaneously and incorporate gene or pathway-level data into the model [56], [57]. Our hierarchical models provide a natural and efficient way to incorporate the external information about candidate genes into the analysis. One way to include the gene-level information in the hierarchical models is to model the prior means of weights and group effects using gene or pathway level predictors [38]. This would allow us to pool the information in the same genes or pathways and thus would provide more effective inference about the genetic effects.

Our fourth extension could incorporate genetic interactions (gene-gene and gene-environment interactions) into the model. Just as interactions must be considered in standard GWA studies [57]–[59], they are also likely to be important in association studies involving rare variants [19]. In principle, we can extend the proposed model to include additional groups for interactions for each pair of groups of main effects and to define an overall effect and a genetic score for each interaction group. However, it would be necessary to investigate statistical power for detecting interactions for rare variants. Finally, we have planned to extend our method to family-based matched case-control association studies. So far the existing methods for rare variants have focused on population-based studies. However, for rare variants, family-based designs may prove very useful [60]. Not only are they robust against population stratification, but they may also offer increased power due to the increased likelihood of affected relatives to share the same rare disease variants. As the conditional logistic regression commonly used for matched case-control studies can be formulated as a Poisson regression [36], our hierarchical generalized linear models can be applied.

The proposed hierarchical generalized linear models may provide efficient tools for disease risk prediction and personalized medicine. GWA studies have raised expectations for predicting individual susceptibility to common diseases using genetic variants [61], [62]. Previous methods using only a limited number of significant variants have typically failed to achieve satisfactory prediction performance [63], [64]. Recent studies show that joint analysis of a large number of genetic variants can improve the prediction of complex traits [65]–[67]. It is understood that a model including as many predictors as possible and fitted appropriately could provide better prediction. Although the previous studies have included many genetic variants in a predictive model, they treat these variants individually and hence could be suboptimal to efficiently use information of genetic variants with small effects and low frequencies. The proposed hierarchical models can better deal with such variants and can integrate external biological knowledge, and therefore may be able to improve the accuracy of prediction.

## Supporting Information

Text S1 The EM-IWLS Algorithm for Fitting Hierarchical Generalized Linear Models.
(DOCX)
