# Supplementary material for: Hierarchical Generalized Linear Models for Multiple Groups of Rare and Common Variants: Jointly Estimating Group and Individual-Variant Effects
Source: PLoS Genet. 2011 Dec 1;7(12):e1002382. doi: 10.1371/journal.pgen.1002382 (PMC3228815; doi:10.1371/journal.pgen.1002382)
Supplement: Text S1 — The EM-IWLS Algorithm for Fitting Hierarchical Generalized Linear Models. (DOCX) [file pgen.1002382.s001.docx]

**Text S1**

**The EM-IWLS Algorithm for Fitting Hierarchical Generalized Linear Models**

We describe our EM-IWLS algorithm for fitting the hierarchical generalized linear models with the linear predictor,

$\eta_{i}=\sum_{j=0}^{J_{0}} x_{ij}\beta_{j}+\sum_{k=1}^{K} \sum_{j\in G_{k}}^{J_{k}} z_{ij}\beta_{j}\triangleq X_{i}\beta$, (S1)

the link function *h*, and the data distribution , where *β*_0_ is the intercept, *x_ij_* and *z_ij_* represent observed values of ungrouped and grouped variables, respectively, *X_i_* contains all variables, and *β* is a vector of all the coefficients and the intercept. For simplicity, we denote $X_{i}=(1,x_{i1},\cdots,x_{iJ})$ and$\beta=(\beta_{0},\beta_{1},\cdots,\beta_{J})'$, where $J=\sum_{g=0}^{K} J_{g}$is the total number of variables. We consider the following prior distributions:

$p(\beta_{0})=N(0,\tau_{0}^{2})$ with $\tau_{0}^{2}$ set to a large value, ,

$\beta_{j}|\tau_{j}^{2}\sim N(\mu_{j}, \tau_{j}^{2})$, $\tau_{j}^{2}|s_{j}^{2}\sim\text{Inv-}\chi^{2}(\nu, s_{j}^{2})$, $s_{j}^{2}|b_{k[j]}\sim\text{Gamma}(a, b_{k[j]})$ (S2)

For grouped variables, $p(\log b_{k})\propto1$

For ungrouped variables, *b_k_*[*_j_*] is a constant

where ν and *a* are constants, the subscript *k*[*j*] indexes the group *k* that the *j*-th predictor belongs to.

We fit the above hierarchical GLMs by incorporating a flexible EM algorithm into the iteratively weighted least squares (IWLS) for fitting classical GLMs. Our EM algorithm treats the unknown variances$\tau_{j}^{2}$ and the hyperparameters $s_{j}^{2}$ and $b_{k[j]}$ as missing data. At each step of the iteration, we replace the terms involving the missing values by their conditional expectations, and then update the parameters () by maximizing the expected value of the joint log-posterior density

(S3)

For the E-step of the algorithm, we take the expectation of the above joint log-posterior density with respect to the conditional posterior distributions,$p(\tau_{j}^{-2}|\beta_{j},s_{j})$, $p(s_{j}^{2}|\tau_{j},b_{k[j]})$ and $p(b_{k[j]}|\{s_{j};j\in G_{k}\})$. With the proposed priors, these conditional posterior distributions can be derived as

$\tau_{j}^{-2}|\beta_{j},s_{j}^{2}\sim\frac{\chi_{1+\nu}^{2}}{\nu s_{j}^{2}+(\beta_{j}-\mu_{j})^{2}}$ (S4)

$s_{j}^{2}|\tau_{j}^{2},b_{k[j]}\sim\text{Gamma}\left(\nu/2+a,\text{ }\tau_{j}^{-2}\nu/2+b_{k[j]}\right)$ (S5)

$b_{k}|\{s_{j}^{2};j\in G_{k}\}\sim\text{Gamma}\left(aJ_{k},\text{ }\sum_{j\in G_{k}}^{J_{k}} s_{j}^{2}\right)$ (S6)

Therefore, we have the conditional expectations

$E(\tau_{j}^{-2}|\beta_{j},s_{j}^{2})=\frac{1+\nu}{\nu s_{j}^{2}+(\beta_{j}-\mu_{j})^{2}}$ (S7)

$E(s_{j}^{2}|\tau_{j}^{2},b_{k[j]})=(\nu/2+a)/(\tau_{j}^{-2}\nu/2+b_{k[j]})$ (S8)

$E(b_{k}|\{s_{j}^{2};j\in G_{k}\})=aJ_{k}/\sum_{j\in G_{k}}^{J_{k}} s_{j}^{2}$ (S9)

Since only the terms $(\beta_{j}-\mu_{j})^{2}/\tau_{j}^{2}$ ($j=1,\cdots,J$) include both the parameters and the missing values, only the conditional expectations $E(\tau_{j}^{-2}|\beta_{j},s_{j})$ directly affect the M-step.

In the M-step, we update () by maximizing , where$\hat{\tau}_{0}^{-2}=\tau_{0}^{-2}$, and $\hat{\tau}_{j}^{-2}=E(\tau_{j}^{-2}|\hat{\beta}_{j},\hat{s}_{j}^{2})$ for $j=1,\cdots,J$. This is equivalent to solving the generalized linear model with the normal priors$\beta_{j}|\hat{\tau}_{j}^{2}\sim N(\mu_{j},\hat{\tau}_{j}^{2})$ [1-3]. Following the usual IWLS algorithm for fitting GLMs (as implemented in the glm function in R), we approximate the generalized linear model likelihood by the weighted normal likelihood

(S10)

where the ‘normal response’ $z_{i}$ and ‘weight’ *w_i_* are called the pseudo-response and pseudo-weight, respectively. The pseudo-response and pseudo-weight are calculated by

$z_{i}=\hat{\eta}_{i}-\frac{L'(y_{i}|\hat{\eta}_{i})}{L''(y_{i}|\hat{\eta}_{i})}, w_{i}=-L''(y_{i}|\hat{\eta}_{i})$ (S11)

where$\hat{\eta}_{i}=X_{i}\hat{\beta}$,,$L'(y_{i}|\eta_{i})=dL(y_{i}|\eta_{i})/d\eta_{i}$, $L''(y_{i}|\eta_{i})=d^{2}L(y_{i}|\eta_{i})/d\eta_{i}^{2}$, and $\hat{\beta}$ is the current estimate of $\beta$.

We then incorporate the prior$\beta_{j}|\hat{\tau}_{j}^{2}\sim N(\mu_{j},\hat{\tau}_{j}^{2})$ into the weighted normal likelihood as an ‘additional data point’ $\mu_{j}$ (the prior mean) with corresponding ‘explanatory variables’ equal to 0 expect *x_j_* which equals 1 and a ‘residual variance’$\hat{\tau}_{j}^{2}$ [1,4]. Therefore, we can update$\beta$ by running the augmented weighted regression

(S12)

where $z_{*}=\left(\begin{matrix} z \\ 0 \end{matrix}\right)_{(n+J+1)\times1}$ is the vector of all $z_{i}$ and all (*J* + 1) prior means 0, $X_{*}=\left(\begin{matrix} X \\ I_{J+1} \end{matrix}\right)_{(n+J+1)\times(J+1)}$ is constructed by the design matrix *X* of the regression and the identity matrix *I_(J+1)_*, and is the diagonal matrix of all pseudo-weights and prior variances. With the augmented$X_{*}$, this regression is identified and thus the resulting estimate$\hat{\beta}$ is well defined and has finite variance, even if the original data are high-dimensional and have collinearity or separation that would result in nonidentifiability of the classical maximum likelihood estimate.

Therefore, we obtain the estimate of $\beta$, $\hat{\beta}=(X_{*}^{'}\Sigma_{*}^{-1}X_{*})^{-1}X_{*}^{'}\Sigma_{*}^{-1}z_{*}$, and its variance

. If a dispersion parameter,, is present, we can update at each step of the iteration by

(S13)

In summary, our EM-IWLS algorithm proceeds as follows:

1. Initialize () with some plausible values. For example, we can set $\beta_{0}$= 0, $\beta_{j}=\mu_{j}$, = 1, $b_{k}$= 0.5, and $s_{j}$ = $\tau_{j}$ = 0.5.
2. For *t* = 1, 2, ··· :

E-step: Calculate the conditional expectations $b_{k}^{(t)}=E(b_{k}|\{s_{j}^{(t-1)};j\in G_{k}\})$, , and .

M-step:

1. Based on the current value of$\beta$, calculate the pseudo-data $z_{i}^{(t)}$ and the pseudo-weights $w_{i}^{(t)}$ using (S11).
2. Update$\beta$ by running the augmented weighted regression (S12).
3. If is present, update using (S13).

We apply the criterion in the glm function to assess convergence, i.e., $\left|d^{(t)}-d^{(t-1)}\right|/\left(0.1+\left|d^{(t)}\right|\right)<\varepsilon$, where $d^{(t)}$ = is the estimate of deviance at the *t*^th^ iteration), and $\varepsilon$ is a small value (say 10^-5^). At convergence of the algorithm, we obtain the latest estimates ($\hat{\beta}$,) and the covariance matrix . As in the classical framework, the *p*-values for testing the hypotheses H_0_: $\beta_{j}=0$ can be calculated using the statistics $\hat{\beta}_{j}/\sqrt{\text{Var(}\hat{\beta}_{j})}$, which approximately follows a standard normal distribution or a Student-*t* distribution with *n* degrees of freedom, if the dispersion is not or in the model, respectively.

**References**

1. Gelman A, Jakulin A, Pittau MG, Su YS (2008) A weakly informative default prior distribution for logistic and other regression models. Annals of Applied Statistics 2: 1360-1383.

2. Yi N, Banerjee S (2009) Hierarchical generalized linear models for multiple quantitative trait locus mapping. Genetics 181: 1101-1113.

3. Yi N, Kaklamani VG, Pasche B (2011) Bayesian analysis of genetic interactions in case-control studies, with application to adiponectin genes and colorectal cancer risk. Ann Hum Genet 75: 90-104.

4. Gelman A, Carlin J, Stern H, Rubin D (2003) Bayesian data analysis. London: Chapman and Hall.
